# Supplementary material for: Targeting Hypoglycemic Natural Products from the Cloud Forest Plants Using Chemotaxonomic Computer-Assisted Selection
Source: Int J Mol Sci. 2024 Oct 10;25(20):10881. doi: 10.3390/ijms252010881 (PMC11507857; doi:10.3390/ijms252010881)
Supplement: Supplementary file 1 [file ijms-25-10881-s001.zip › ijms-3218489-supplementary.pdf]

S1. Metabolic pathways identified in *S. rhombifolia* and *S. glabra*.

Pathway*	Total	Expected	Hits	*p-value*	$-LOG_{10}(P)$	Holm adjust	FDR	Impact
Flavonoid biosynthesis	47	3.1376	20	1.0412e-12	11.982	9.9959e-11	5.4368e-11	0.37763
Flavone and flavonol biosynthesis	10	0.66757	10	1.1327e-12	11.946	1.076e-10	5.4368e-11	1
Galactose metabolism	27	1.8024	15	7.3364e-12	11.135	6.8962e-10	2.3476e-10	0.78733
Phenylpropanoid biosynthesis	46	3.0708	13	3.8615e-06	5.4132	0.00035912	9.2676e-05	0.33266
Biosynthesis of secondary metabolites - other antibiotics	6	0.40054	4	0.00025317	3.5966	0.023291	0.0048608	0
Starch and sucrose metabolism	22	1.4686	7	0.00035552	3.4491	0.032353	0.0056884	0.51891
Linoleic acid metabolism	4	0.26703	3	0.0011002	2.9585	0.099022	0.015089	1
Diterpenoid biosynthesis	28	1.8692	6	0.0085966	2.0657	0.7651	0.091697	0.24243
alpha-Linolenic acid metabolism	28	1.8692	6	0.0085966	2.0657	0.7651	0.091697	0.38331
Cutin, suberine, and wax biosynthesis	18	1.2016	4	0.027669	1.558	1	0.26562	0.125
Fructose and mannose metabolism	20	1.3351	4	0.039542	1.4029	1	0.34509	0.10854
Stilbenoid, diarylheptanoid, and gingerol biosynthesis	8	0.53405	2	0.0949	1.0227	1	0.7592	0.2647
Biosynthesis of unsaturated fatty acids	22	1.4686	3	0.17742	0.75099	1	1	0
Indole alkaloid biosynthesis	4	0.26703	1	0.24168	0.61676	1	1	0
Biosynthesis of secondary metabolites - unclassified	5	0.33378	1	0.29244	0.53397	1	1	1
Isoquinoline alkaloid biosynthesis	6	0.40054	1	0.33983	0.46874	1	1	0
Ascorbate and aldarate metabolism	18	1.2016	2	0.3407	0.46763	1	1	0.1791
Porphyrin and chlorophyll metabolism	48	3.2043	4	0.40046	0.39744	1	1	0.2891
Tropane, piperidine, and pyridine alkaloid biosynthesis	8	0.53405	1	0.42539	0.37121	1	1	0
Amino sugar and nucleotide sugar metabolism	50	3.3378	4	0.43159	0.36493	1	1	0
Phenylalanine, tyrosine, and tryptophan biosynthesis	22	1.4686	2	0.43814	0.35839	1	1	0.00851
Glycolysis / Gluconeogenesis	26	1.7357	2	0.52741	0.27785	1	1	0.00114
Riboflavin metabolism	11	0.73432	1	0.53357	0.27281	1	1	0.11852
Phenylalanine metabolism	11	0.73432	1	0.53357	0.27281	1	1	0.23529
Cyanoamino acid metabolism	29	1.9359	2	0.5879	0.2307	1	1	0.11864
Terpenoid backbone biosynthesis	30	2.0027	2	0.60676	0.21698	1	1	0.11971
Tyrosine metabolism	16	1.0681	1	0.67086	0.17337	1	1	0.14189
Ubiquinone and other terpenoid-quinone biosynthesis	38	2.5367	2	0.73467	0.13391	1	1	0.02324
Phosphatidylinositol signaling system	26	1.7357	1	0.8367	0.077429	1	1	0.03285
Folate biosynthesis	27	1.8024	1	0.8478	0.071707	1	1	0
Inositol phosphate metabolism	28	1.8692	1	0.85815	0.066438	1	1	0.10251
Arginine and proline metabolism	34	2.2697	1	0.90713	0.042332	1	1	0.0122
Pyrimidine metabolism	38	2.5367	1	0.93004	0.031496	1	1	0.07921

*The identification was made using the total rt-*m/z* database in both ionization modes (ESI+ and ESI-), the Mummichog algorithm, the KEGG database, and the *Arabidopsis thaliana* library.
FDR= False Discovery Rate

S2. Putative annotation by LC-ESI-HRMS/MS of natural products in the methanolic extract of *S. rhombifolia*.

Metabolite	Retention time	Precursor Ion (*m/z*)	Adduct	Formula	Fragment Ions
Corchoionoside C	4.16	409.1822	$[M+Na]^+$	$C_{19}H_{30}O_8$	257.1021 ($C_{12}H_{17}O_6$), 233.1025 ($C_{10}H_{17}O_6$)
cis-Tiliroside	6.79	595.1442	$[M+H]^+$	$C_{30}H_{26}O_{13}$	309.0993 ($C_{15}H_{17}O_7$), 287.0549 ($C_{15}H_{11}O_6$)
Kaempferitrin	4.41	577.1547	$[M-H]^-$	$C_{27}H_{30}O_{14}$	431.0986 ($C_{21}H_{19}O_{10}$), 413.0864 ($C_{21}H_{17}O_9$)
Cleomiscosin A	7.82	385.0916	$[M-H]^-$	$C_{20}H_{18}O_8$	367.0819 ($C_{20}H_{15}O_7$), 308.0669 ($C_{18}H_{12}O_5$)
Cappariloside A	4.44	333.1091	$[M-H]^-$	$C_{16}H_{18}N_2O_6$	289.1176 ($C_{15}H_{17}N_2O_4$), 249.0664 ($C_{15}H_9N_2O_2$)
trans-Tiliroside	6.55	617.1265	$[M+Na]^+$	$C_{30}H_{26}O_{13}$	291.0876 ($C_{15}H_{15}O_6$), 287.0557 ($C_{15}H_{11}O_6$)
Cleomiscosin B	6.66	385.0924	$[M-H]^-$	$C_{20}H_{18}O_8$	353.0652 ($C_{19}H_{13}O_7$), 294.0536 ($C_{17}H_{10}O_5$)
Depressonol A	3.88	741.1872	$[M-H]^-$	$C_{32}H_{38}O_{20}$	487.1213 ($C_{24}H_{23}O_{11}$), 475.1061 ($C_{19}H_{23}O_{14}$)
Isolimonic acid glucoside*	5.48	667.2601	$[M-H]^-$	$C_{32}H_{44}O_{15}$	583.2195 ($C_{31}H_{35}O_{11}$), 440.11 ($C_{23}H_{20}O_9$)
Ichangic acid 17-β-D-glucopyranoside*	5.48	667.2601	$[M-H]^-$	$C_{32}H_{44}O_{15}$	583.2195 ($C_{31}H_{35}O_{11}$), 559.2751 ($C_{27}H_{43}O_{12}$)
Citrusin E	5.85	371.135	$[M-H]^-$	$C_{17}H_{24}O_9$	341.1246 ($C_{16}H_{21}O_8$), 179.071 ($C_{10}H_{11}O_3$)
Rhaponticin	4.85	419.1338	$[M-H]^-$	$C_{21}H_{24}O_9$	401.1232 ($C_{21}H_{21}O_8$)
20-Hydroxyecdysone	5.8	515.2773	$[M+Cl]^-$	$C_{27}H_{44}O_7$	479.3007 ($C_{27}H_{43}O_7$)
Acanthoside D	4.27	741.2592	$[M-H]^-$	$C_{34}H_{46}O_{18}$	417.1543 ($C_{22}H_{25}O_8$), 179.0549 ($C_6H_{11}O_6$)
Cleomiscosin D	7.63	415.1025	$[M-H]^-$	$C_{21}H_{20}O_9$	237.0754 ($C_{12}H_{13}O_5$), 177.0182 ($C_9H_5O_4$)
Thermopsoside	4.12	461.1094	$[M-H]^-$	$C_{22}H_{22}O_{11}$	300.0262 ($C_{15}H_8O_7$), 271.0231 ($C_{14}H_7O_6$)
Ferulic acid	3.39	193.0497	$[M-H]^-$	$C_{10}H_{10}O_4$	178.0272 ($C_9H_6O_4$), 149.0607($C_9H_9O_2$)
1-*O*-Sinapoyl-β-D-glucose	4.26	385.1135	$[M-H]^-$	$C_{17}H_{22}O_{10}$	181.0494 ($C_9H_9O_4$), 163.0396 ($C_9H_7O_3$)
Neoacrimarine E	13.59	612.2245	$[M-H]^-$	$C_{35}H_{35}NO_9$	379.1794 ($C_{23}H_{25}NO_4$), 312.1715 ($C_{20}H_{24}O_3$)
Polypodine B	4.56	531.2717	$[M+Cl]^-$	$C_{30}H_{48}O_8$	495.2958 ($C_{27}H_{44}O_8$)
Corchorusoside D*	12.49	699.3564	$[M+H]^+$	$C_{35}H_{54}O_{14}$	659.3619 ($C_{33}H_{55}O_{13}$), 537.3041 ($C_{29}H_{45}O_9$)
Corchorusoside A*	12.49	699.3564	$[M+H]^+$	$C_{35}H_{54}O_{14}$	659.3619 ($C_{33}H_{55}O_{13}$), 537.3041 ($C_{29}H_{45}O_9$)

*Undistinguished isomers

S3. Binding free energy between the metabolites identified in the methanolic extract of *S. rhombifolia* and the DMII molecular targets.

Metabolite	DPP-IV		αA		αG	
	Mean	SD	Mean	SD	Mean	SD
trans-Tiliroside	-11.2	0.57	-11.4	0.30	-10.8	1.60
cis-Tiliroside	-11.1	0.59	-11.4	0.45	-10.9	1.54
Depressonol A	-10.2	0.47	-10.4	0.21	-9.8	1.53
Naringin	-9.5	0.37	-9.5	0.34	-9.1	1.12
Kaempferitrin	-9.5	0.58	-9.8	0.68	-8.6	1.29
Acanthoside D	-9.5	0.37	-9.2	0.33	-8.7	0.81
Thermopsoside	-9.2	0.43	-9.7	0.78	-9.0	0.71
Kuromanin	-9.1	0.54	-9.4	0.21	-8.7	0.90
Corchorusoside D	-8.8	0.33	-9.1	0.56	-8.4	0.90
Rhaponticin	-8.6	0.51	-9.4	0.60	-9.0	0.81
Corchorusoside A	-8.5	0.32	-8.5	0.34	-8.3	0.80
Ichangic acid 17-beta-D-glucopyranoside	-8.2	0.60	-7.6	0.30	-7.9	1.05
Isolimonic acid glucoside	-8.1	0.43	-8.1	0.37	-8.4	1.09
Neoacrimarine E	-7.9	0.43	-8.4	0.53	-7.7	1.77
Cleomiscosin B	-7.8	0.33	-8.0	0.48	-7.1	0.92
Cleomiscosin D	-7.7	0.21	-8.1	0.57	-7.1	1.09
Polypodine B	-7.6	0.25	-7.9	0.10	-7.6	0.94
1-O-Sinapoyl-beta-D-glucose	-7.5	0.26	-7.8	0.43	-7.9	0.35
20-Hydroxyecdysone	-7.4	0.36	-7.7	0.28	-7.5	0.84
Cleomiscosin A	-7.4	0.35	-7.8	0.74	-6.8	1.00
Citrusin E	-7.2	0.17	-7.4	0.52	-7.5	0.53
Cappariloside A	-7.1	0.24	-6.9	0.27	-7.1	0.52
Corchoionoside C	-6.7	0.16	-7.0	0.22	-7.1	0.31
Ferulic acid	-5.6	0.46	-5.8	0.13	-5.8	0.46
Vanillic acid	-5.2	0.43	-5.2	0.45	-5.5	0.48
trans-Cinnamic acid	-4.9	0.26	-5.4	0.32	-5.3	0.47
Vanillin	-4.8	0.23	-4.8	0.43	-5.0	0.40
Metformin	-3.5	0.17	-3.5	0.30	-4.0	0.27
Sitagliptin	-8.0	0.25	-8.4	0.68	-8.5	0.34
Acarbose	-8.2	0.17	-8.1	0.18	-8.5	0.41

**Data are expressed as mean binding free energy (kcal/mol) of the replicates of each compound (orientations: n=5) and the conformers of each enzyme (n=6) ± standard deviation.

S4. Binding modes (left) and intermolecular interactions diagrams (right) of ten selected metabolites tentatively identified in *S. rhombifolia*, in complex with DPP-IV.

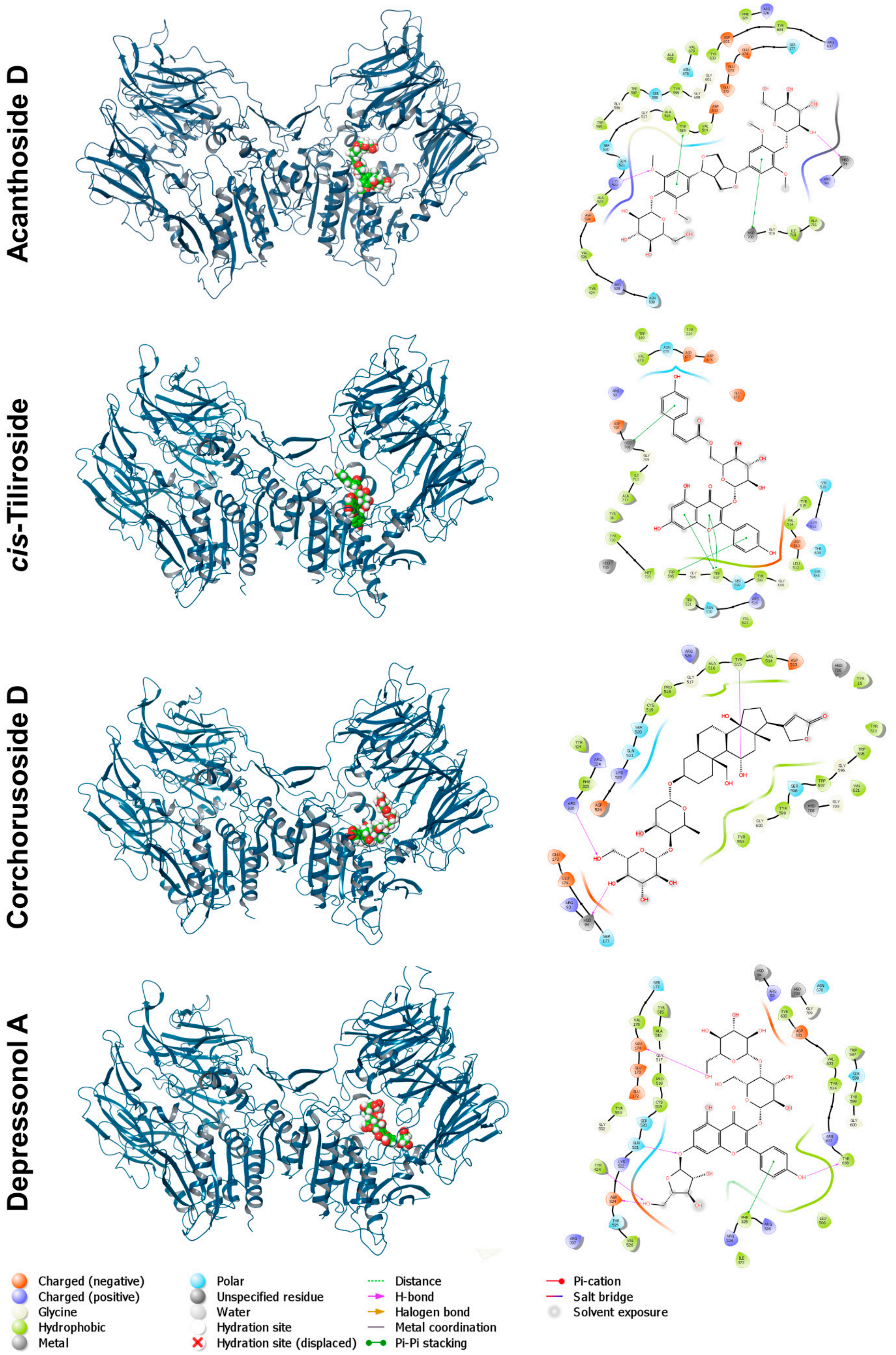

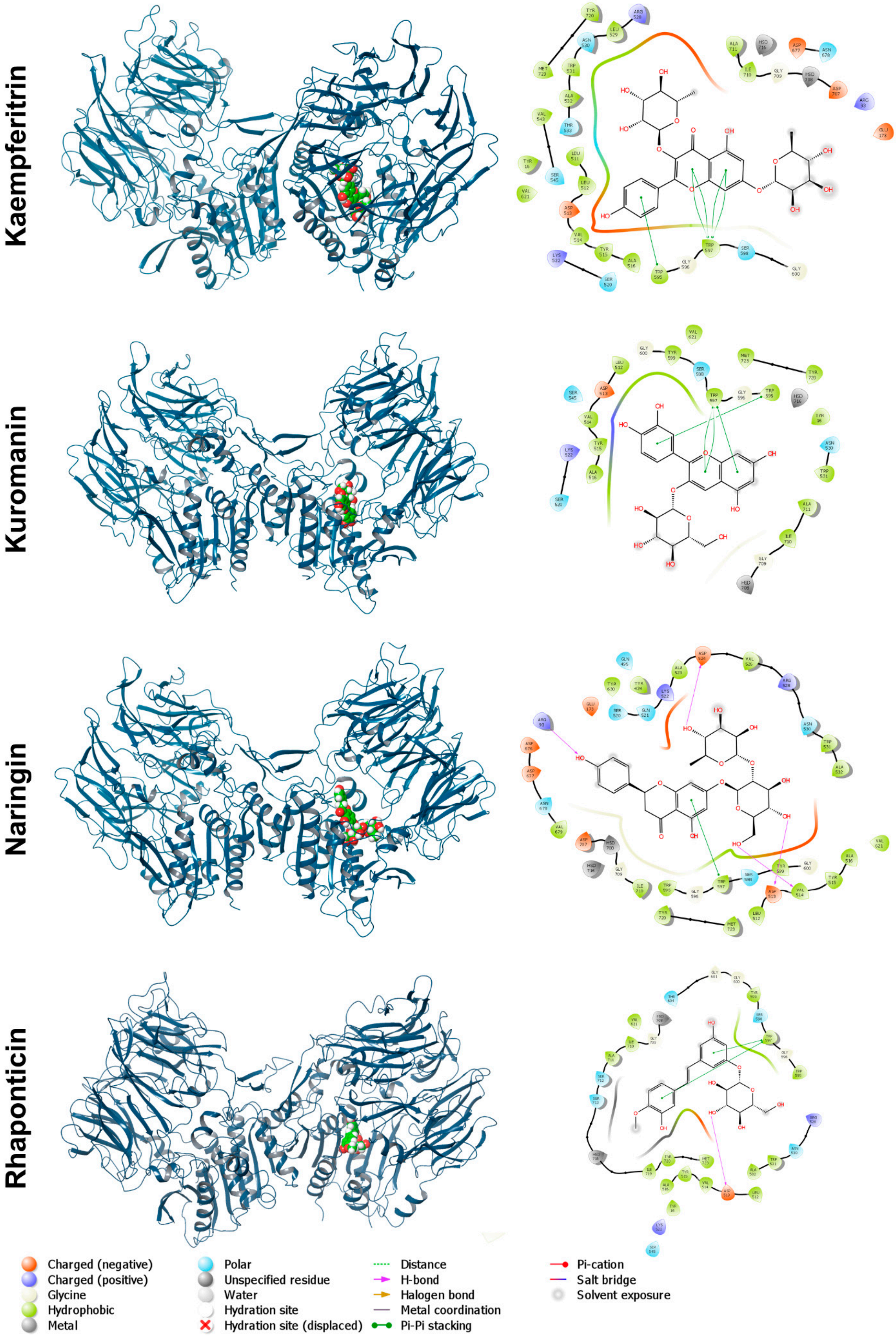
Kaempferitrin
Kuromanin
Naringin
Rhaponticin
Charged (negative)
Charged (positive)
Glycine
Hydrophobic
Metal
Polar
Unspecified residue
Water
Hydration site
Hydration site (displaced)
Distance
H-bond
Halogen bond
Metal coordination
Pi-Pi stacking
Pi-cation
Salt bridge
Solvent exposure

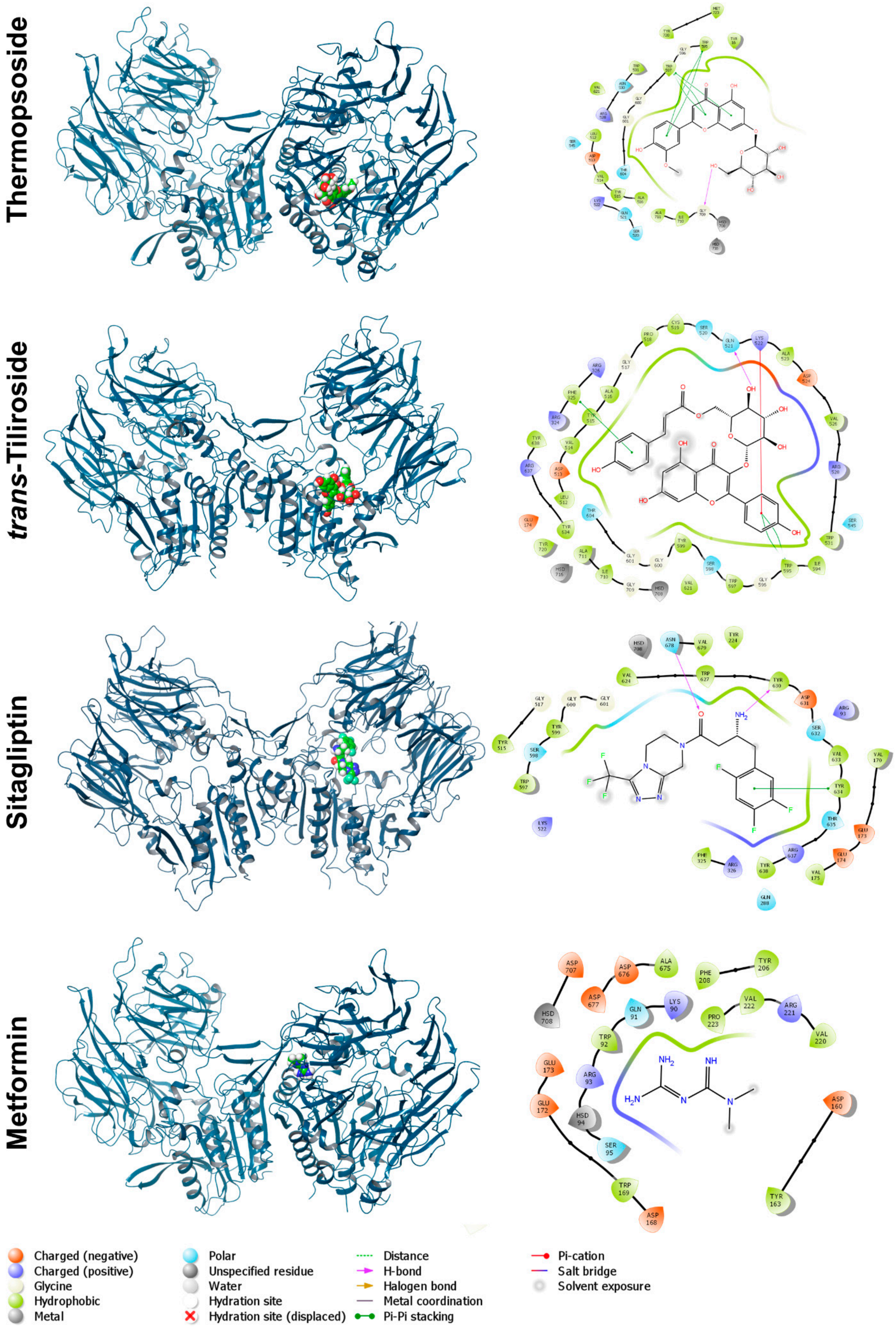
Thermopsoside
trans-Tiliroside
Sitagliptin
Metformin
Charged (negative)
Charged (positive)
Glycine
Hydrophobic
Metal
Polar
Unspecified residue
Water
Hydration site
Hydration site (displaced)
Distance
H-bond
Halogen bond
Metal coordination
Pi-Pi stacking
Pi-cation
Salt bridge
Solvent exposure

S5. Binding modes (left) and intermolecular interactions diagrams (right) of ten selected metabolites tentatively identified in *S. rhombifolia*, in complex with αA.

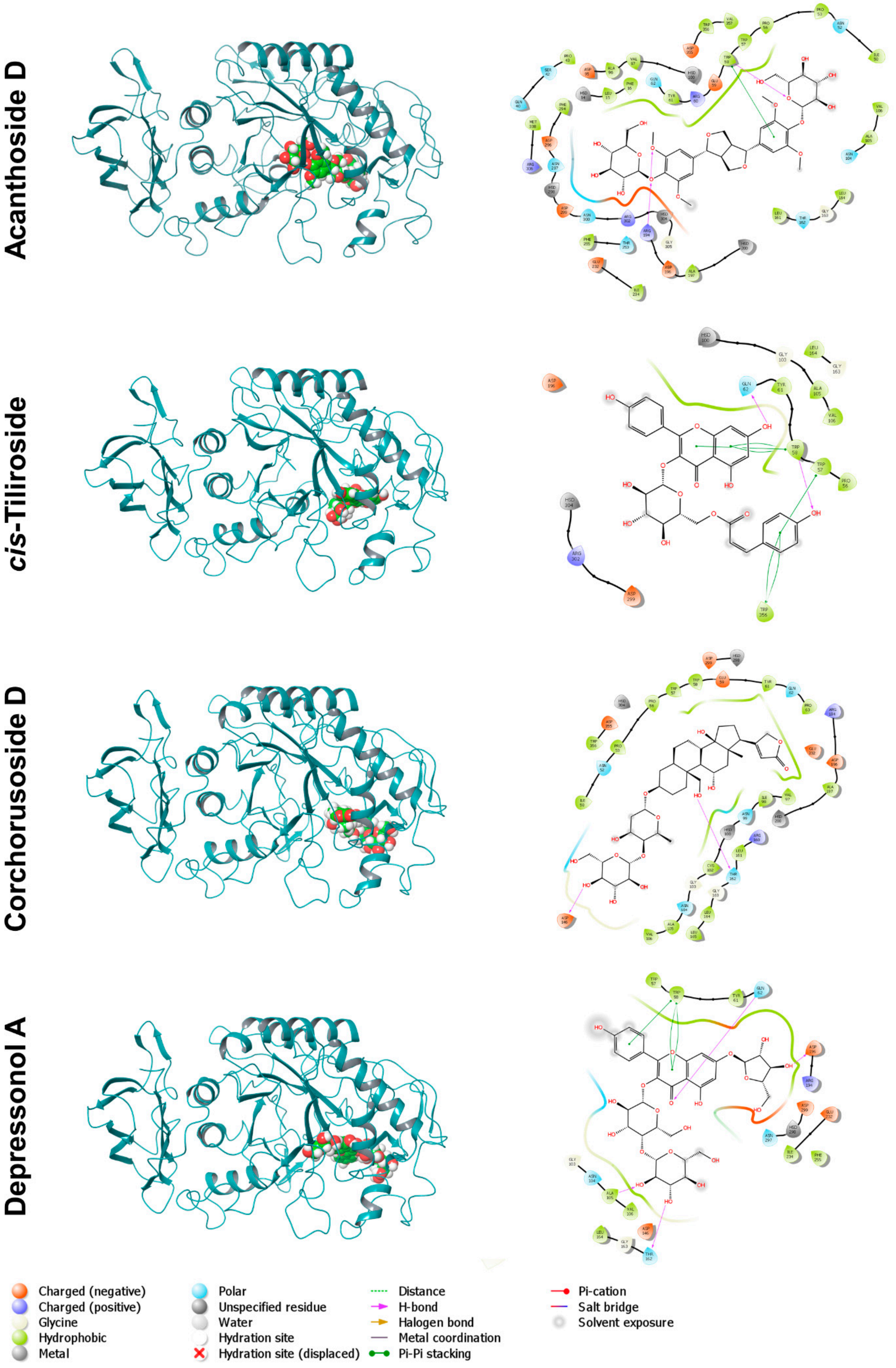

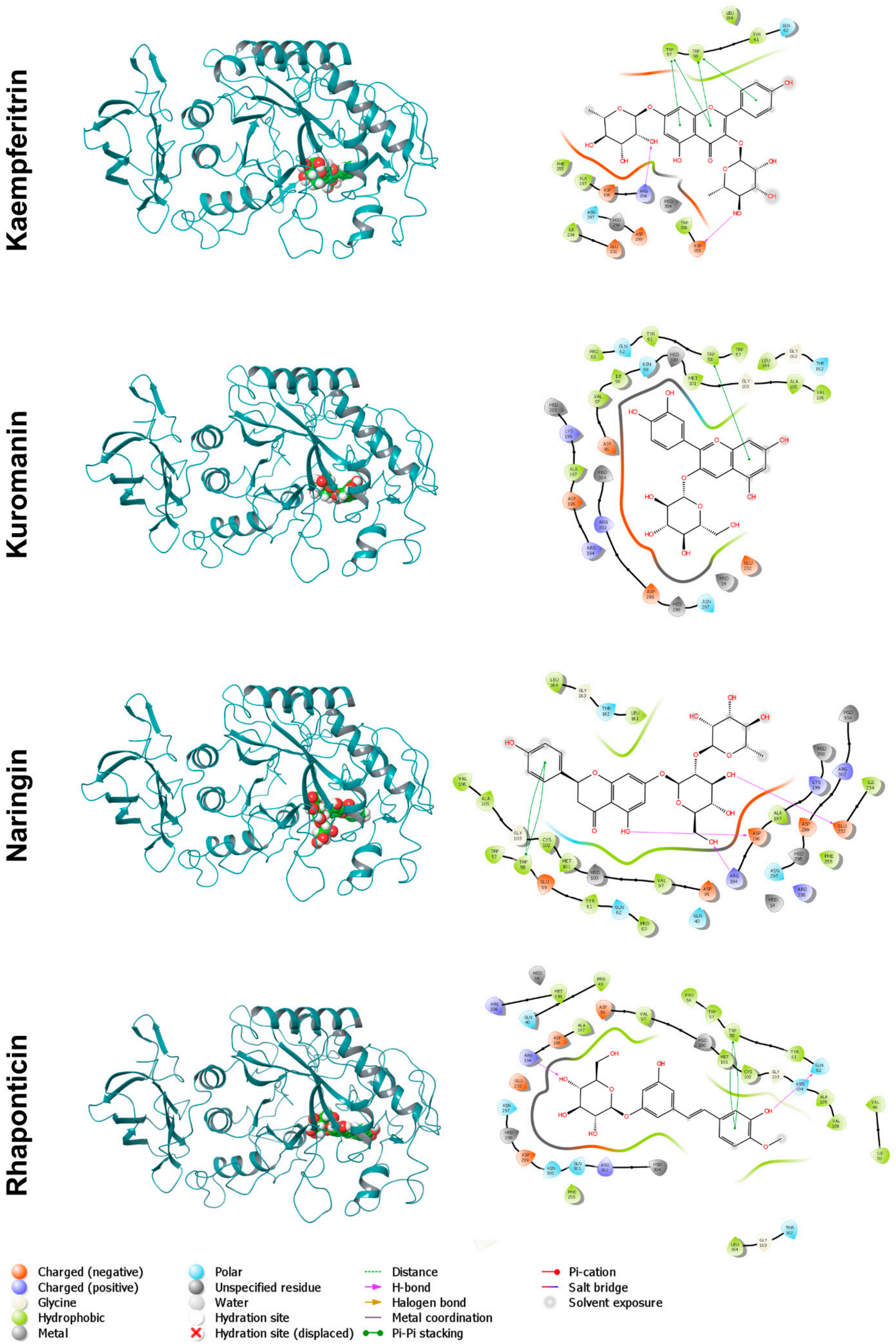
Kaempferitrin
Kuromanin
Naringin
Rhaponticin
Charged (negative)
Charged (positive)
Glycine
Hydrophobic
Metal
Polar
Unspecified residue
Water
Hydration site
Hydration site (displaced)
Distance
H-bond
Halogen bond
Metal coordination
Pi-Pi stacking
Pi-cation
Salt bridge
Solvent exposure

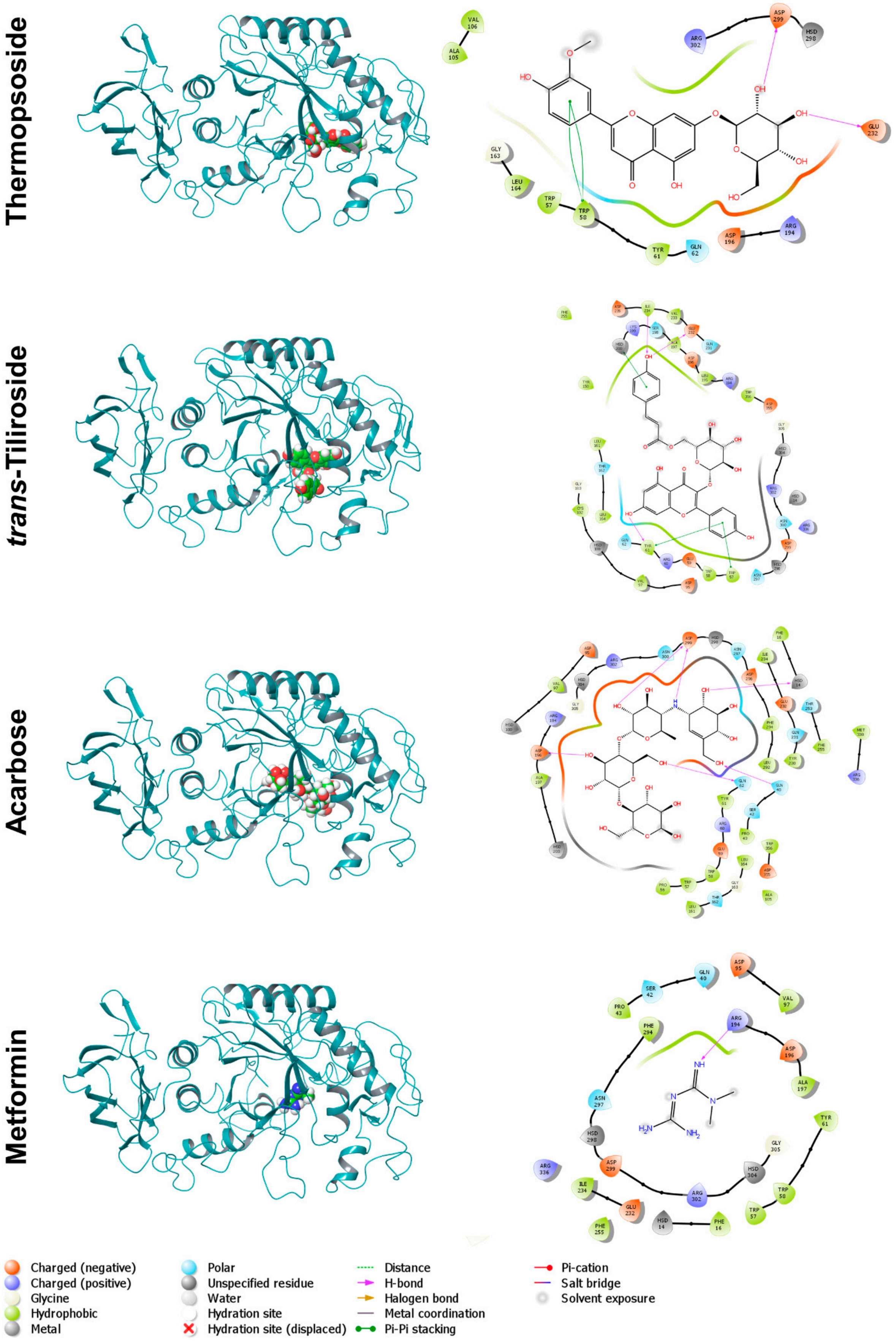

Thermopsoside
trans-Tiliroside
Acarbose
Metformin
Charged (negative)
Charged (positive)
Glycine
Hydrophobic
Metal
Polar
Unspecified residue
Water
Hydration site
Hydration site (displaced)
Distance
H-bond
Halogen bond
Metal coordination
Pi-Pi stacking
Pi-cation
Salt bridge
Solvent exposure

S6. Binding modes (left) and intermolecular interactions diagrams (right) of ten selected metabolites tentatively identified in *S. rhombifolia*, in complex with αG.

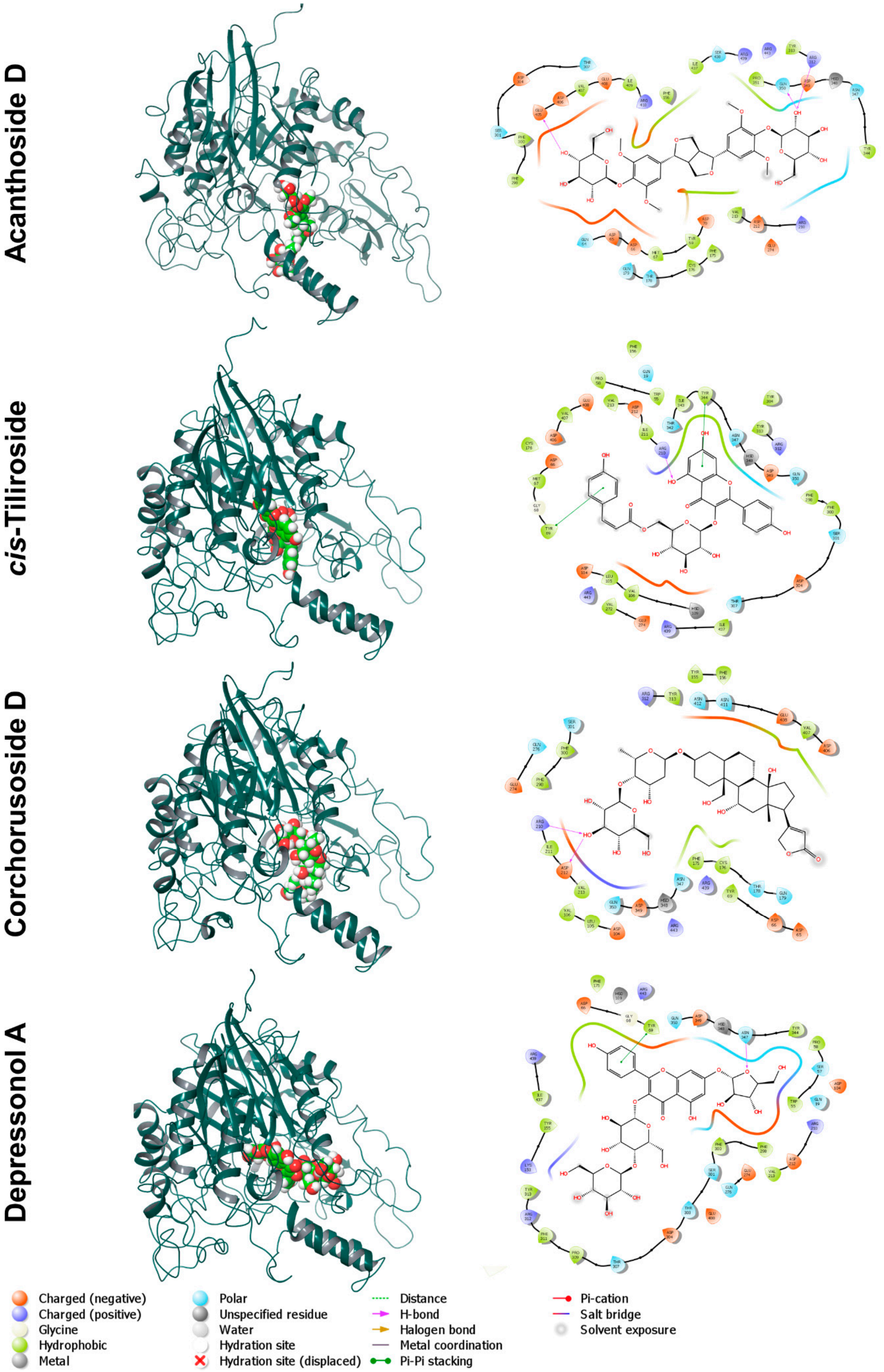

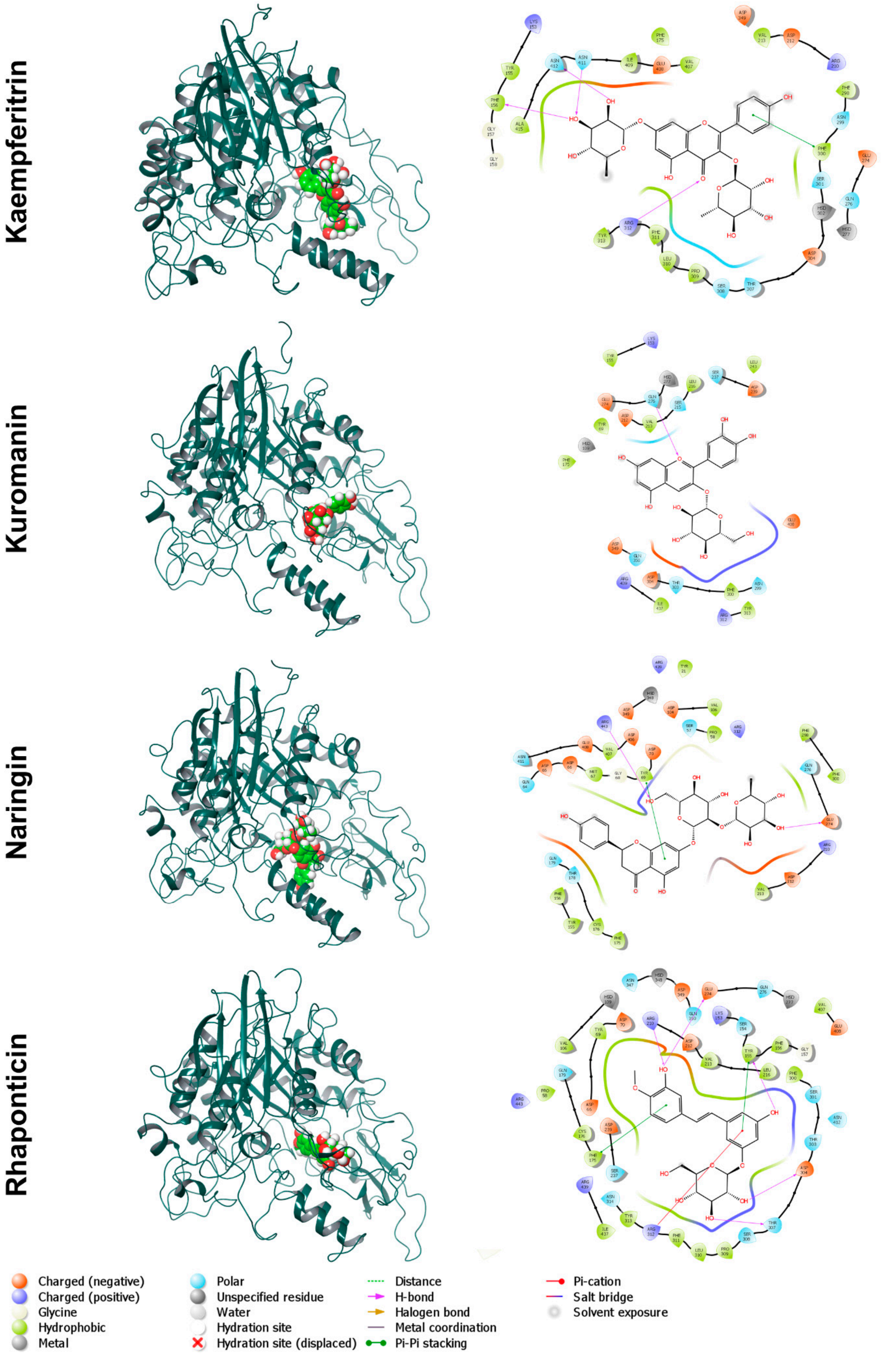
Kaempferitrin
Kuromanin
Naringin
Rhaponticin
Charged (negative)
Charged (positive)
Glycine
Hydrophobic
Metal
Polar
Unspecified residue
Water
Hydration site
Hydration site (displaced)
Distance
H-bond
Halogen bond
Metal coordination
Pi-Pi stacking
Pi-cation
Salt bridge
Solvent exposure

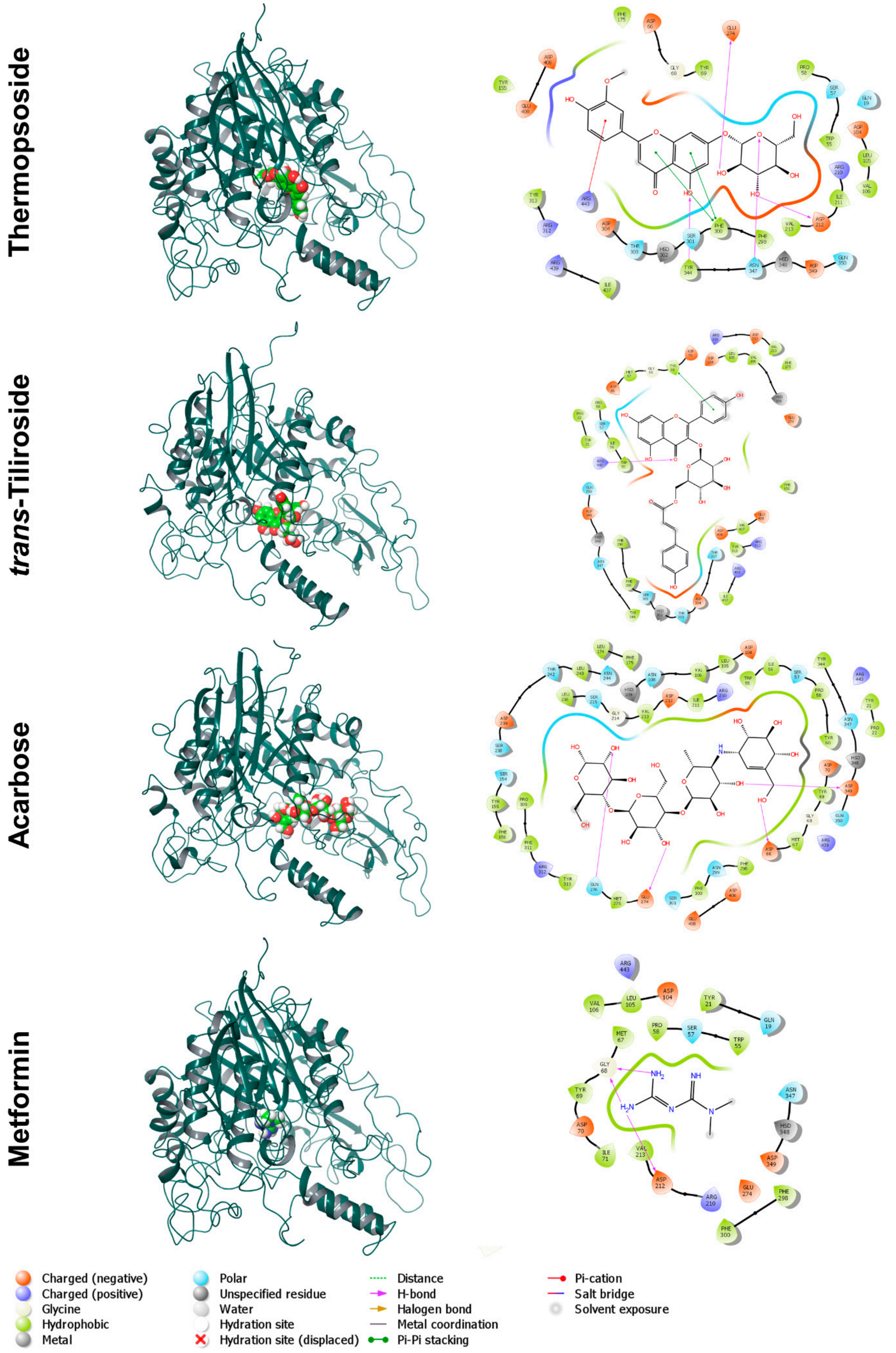
Thermopsoside
trans-Tiliroside
Acarbose
Metformin
Charged (negative)
Charged (positive)
Glycine
Hydrophobic
Metal
Polar
Unspecified residue
Water
Hydration site
Hydration site (displaced)
Distance
H-bond
Halogen bond
Metal coordination
Pi-Pi stacking
Pi-cation
Salt bridge
Solvent exposure

S7. UPLC-ESI-HRMS/MS conditions for untargeted metabolomic analysis.

The analysis was performed on an ultra-high-performance liquid chromatography system (Acquity UPLC I Class, Waters, USA) coupled to electrospray ionization source-single quadrupole-time of flight mass spectrometer (Q-TOF; Synapt G2 Si, Waters, UK). The separation was carried out in an Acquity BEH column (2.1 mm x 50 mm, 1.7 µm, C18, Waters, UK). The column oven and sample manager temperatures were 40 °C and 15 °C, respectively. The flow rate was 300 µL/min, and 5 µL of each sample was injected into the UPLC-ESI-HRMS system in triplicate. The mobile phase consisted of water (A) and acetonitrile (B), both acidified with formic acid (0.1% v/v), and the elution gradient conditions were as follows:

**Time (min)**	**% A**	**% B**
0	99	1
13	20	80
14	20	80
15	99	1
20	99	1

The detection was carried out in both positive and negative ionization modes with the following parameters:

**Parameter**	**Value**
Capillary voltage	3 kV
Capillary temperature	100 °C
Desolvatation gas flow (N_2)	600 L/h
Desolvatation gas temperature	200 °C
Sampling cone voltage	40 V
Source offset voltage	80 V
Nebulizer pressure	6.5 Bar

The mass spectra were acquired in a mass-to-charge ratio range of 50-1200 Da and a scan time of 0.5 s [function 1 (low collision energy) = 6 V; function 2 (high collision energy) = 10-30 V]. The real-time mass exact correction was done with the reference peptide Leucine-enkephalin ($[M+H]^+$=556.2771; ($[M-H]^-$=554.2615). Data management was carried out with MassLynx (V. 4.1, Waters, UK) and MarkerLynx (V 4.1, Waters, UK).

S8. UPLC-ESI-MS/MS conditions for phenolics-targeted metabolomic analysis.

The analysis was performed on an ultra-high-performance liquid chromatography system (1290 Infinity series, Agilent Technologies, USA) coupled to an electrospray ionization source-triple quadrupole mass spectrometer (QqQ; 6460, Agilent Technologies, USA). The separation was carried out in an Acquity BEH column (2.1 mm x 50 mm, 1.7 µm, C18, Waters, U). The temperature for the column oven was 40 °C. The flow rate was 300 µL/min, and 2 µL of each sample was injected into the UPLC-ESI-MS system in triplicate. The mobile phase consisted of water (A) and acetonitrile (B), both acidified with formic acid (0.1% v/v), and the elution gradient conditions were as follows:

Time (min)	A (%)	B (%)
0	99	1
30	50	50
35	1	99
39	1	99
40	99	1
45	99	1

The detection was carried out in both positive and negative ionization modes based on the analyte, with the following parameters:

Parameter	Value
Gas temperature	300º C
Gas flow	5 L/min
Nebulizer pression	45 psi
Sheath Gas Temp	250 º C
Sheath Gas Flow	11 L/min
Capillary voltage (positive and negative)	3.5 kV
Nozzle voltage (positive and negative)	500 V

The protocol used was a dynamic MRM (Multiple Reaction Monitoring). The conditions for each compound are described in the following table:

Compound	dMRM transition			Mass spectrometric conditions			Quantification conditions		
	Precursor ion	Fragment ion	Retention time	Collision energy	Fragmentor	Polarity	Quantification range (µM)	Regression type	R^2
Shikimic acid	173.1	111.1	0.54	10	100	Negative	0.5 - 19	Quadratic	0.97
Gallic acid	169.0	125.2	1.35	10	100	Negative	0.5 - 19	Quadratic	0.99
L-Phenylalanine	166.1	131.0	2.23	10	100	Positive	0.5 - 19	Quadratic	0.99
Protocatechuic acid	153.0	109.1	2.54	10	100	Negative	0.5 - 19	Quadratic	0.99
Gentisic acid	153.0	109.0	3.79	10	100	Negative	0.5 - 19	Quadratic	0.98
4-Hydroxybenzoic acid	137.1	92.8	3.83	10	100	Negative	0.5 - 19	Quadratic	0.99
(-)-Epigallocatechin	305.1	125.0	4.62	20	140	Negative	0.5 - 19	Quadratic	0.99
4-Hydroxyphenylacetic acid	107.1	77.0	4.94	20	140	Positive	0.5 - 19	Quadratic	0.99
(+)-Catechin	291.0	138.9	4.96	10	100	Positive	0.25 - 19	Quadratic	0.99
Vanillic acid	169.0	93.0	5.17	10	100	Positive	0.5 - 19	Quadratic	0.99
Chlorogenic acid	355.1	163.0	5.23	10	100	Positive	0.25 - 19	Quadratic	0.99
Caffeic acid	181.0	163.0	5.31	10	100	Positive	0.25 - 19	Quadratic	0.99
Malvin	655.1	331.1	5.63	40	100	Positive	0.5 - 19	Quadratic	0.99
Kuromanin	449.0	286.9	6.07	30	100	Positive	0.25 - 19	Quadratic	0.99
Procyanidin B2	577.1	425.1	6.21	10	100	Negative	0.5 - 19	Quadratic	0.99
Keracyanin	595.2	287.1	6.57	20	100	Positive	0.125 - 19	Quadratic	0.99
Vanillin	153.0	124.9	6.65	10	100	Positive	0.25 - 19	Quadratic	0.99
(-)-Epicatechin	291.0	138.8	6.79	10	100	Positive	0.5 - 19	Quadratic	0.99
Mangiferin	423.0	302.8	6.90	10	100	Positive	0.5 - 19	Quadratic	0.99
4-Coumaric acid	165.0	147.0	7.15	10	100	Positive	0.5 - 19	Quadratic	0.99
(-)-Gallocatechin gallate	458.9	139.0	7.57	20	80	Positive	0.5 - 19	Quadratic	0.98
Umbelliferone	163.0	107.0	7.65	30	100	Positive	0.5 - 19	Quadratic	0.99
Scopoletin	193.0	133.0	8.30	10	100	Positive	0.5 - 19	Quadratic	0.99
Quercetin 3,4-di-O-glucoside	627.0	302.9	8.40	10	100	Positive	0.5 - 19	Quadratic	0.98
Ferulic acid	195.1	145.0	8.53	20	100	Positive	0.5 – 19	Quadratic	0.99
3-Coumaric acid	165.05	147.04	8.96	10	100	Positive	0.5 – 19	Quadratic	0.99
Sinapic acid	225.1	207.1	8.97	10	100	Positive	0.25 - 19	Quadratic	0.99
Ellagic acid	300.5	145.0	9.25	30	170	Negative	0.5 - 19	Quadratic	0.97
Salicylic acid	137.0	93	9.51	10	100	Negative	0.5 - 19	Quadratic	0.98
Myricitrin	465.0	318.9	9.61	10	100	Positive	0.5 – 19	Quadratic	0.99

Epicatechin gallate	443.1	123.0	9.65	10	100	Positive	0.5 - 19	Quadratic	0.99
Quercetin 3-D-galactoside	465.0	302.9	9.82	10	100	Positive	0.5 - 19	Quadratic	0.99
Rutin	611.0	302.9	9.92	10	100	Positive	0.5 - 19	Quadratic	0.97
Quercetin 3-glucoside	465.0	303.0	10.13	10	100	Positive	0.5 - 19	Quadratic	0.99
Luteolin 7-O-glucoside	449.0	287.0	10.44	10	100	Positive	0.5 – 19	Quadratic	0.98
p-Anisic acid	153.1	109.0	10.8	5	120	Positive	0.25 - 19	Quadratic	0.99
Penta-O-galloyl-B-D-glucose	771.1	153.0	11.30	20	100	Positive	0.25 - 19	Quadratic	0.99
Kaemperol 3-O-glucoside	449.0	286.9	11.47	10	100	Positive	0.5 - 19	Quadratic	0.98
Quercitrin	449.1	303.1	11.54	10	100	Positive	0.5 - 19	Quadratic	0.99
2,4-Dimethoxy-6-methylbenzoic acid	197.0	179.0	11.62	5	80	Positive	0.25 - 19	Quadratic	0.99
Myricetin	317.0	179.0	11.70	10	100	Negative	0.5 - 19	Quadratic	0.96
Naringin	273.0	153.0	12.09	10	120	Positive	0.5 - 19	Quadratic	0.99
trans-Resveratrol	229.1	135.0	12.29	10	100	Positive	0.125 - 19	Quadratic	0.99
Rosmarinic acid	361.1	163.0	12.54	10	100	Positive	0.25 - 19	Quadratic	0.99
Hesperidin	609.1	301.1	12.66	20	100	Negative	0.5 - 19	Quadratic	0.99
Secoisolariciresinol	363.2	137.1	12.82	20	100	Positive	0.5 - 19	Quadratic	0.99
Phloridzin	435.0	272.9	13.02	10	100	Negative	0.5 - 19	Quadratic	0.99
trans-Cinnamic acid	149.1	131.0	14.42	10	100	Positive	0.25 - 19	Quadratic	0.99
Quercetin	301	151	14.67	20	100	Negative	0.125 - 19	Quadratic	0.99
Luteolin	285	151	14.75	20	100	Negative	0.5 - 19	Quadratic	0.99
Psoralen	187.0	131.1	14.78	20	100	Positive	0.5 - 19	Quadratic	0.99
Angelicin	187.0	131.1	15.54	20	100	Positive	0.25 - 19	Quadratic	0.99
Naringenin	271.0	151	16.47	10	100	Negative	0.5 - 19	Quadratic	0.99
Apigenin	271.0	153.0	16.86	30	100	Positive	0.25 - 19	Quadratic	0.99
Kaempferol	285	151	17.27	10	100	Negative	0.5 - 19	Quadratic	0.98
Hesperetin	303.1	177.1	17.7	20	100	Positive	0.25 - 19	Quadratic	0.99
Podophyllotoxin	415.1	397.1	18.8	10	100	Positive	0.25 - 19	Quadratic	0.99
Methyl cinnamate	163.1	131.0	21.34	6	100	Positive	0.25 - 19	Quadratic	0.99
Chrysin	255.1	153.0	22.6	40	100	Positive	0.25 - 19	Quadratic	0.99
Nordihydroguaiaretic acid	303.0	193.1	22.81	10	100	Positive	0.5 - 19	Quadratic	0.93
Kaempferide	299	284	23.98	20	100	Positive	0.5 - 19	Quadratic	0.99
Emodin	269.0	225.0	27.26	20	150	Negative	0.5 - 19	Quadratic	0.99
Chrysophanol	255.1	153.0	30.98	40	100	Positive	0.5 - 19	Quadratic	0.98

The retention time variation allowed for the search of the compounds was 2 min in each case. The cell accelerator voltage was 7 V for each compound. If the concentration of some compounds was higher than the linearity range, dilutions were made.
